# Accelerating Point Cloud Computation via Memory in Embedded Structured Light Cameras

**DOI:** 10.3390/jimaging12020091

**Published:** 2026-02-21

**Authors:** Yanan Zhang, Shikang Meng, Shijie Wang, Yaheng Ren

**Affiliations:** 1 Institute of Applied Mathematics, Hebei Academy of Sciences, Shijiazhuang 050081, China; zhangyanan@heb-as.com (Y.Z.); mengshikang@heb-as.com (S.M.); wangshijie@heb-as.com (S.W.); 2 Hebei Information Security Certification Technology Innovation Center, Shijiazhuang 050081, China

**Keywords:** 3D reconstruction, point cloud computation, parameter calibration, structured light camera, parameter calibration

## Abstract

Embedded structured light cameras have been widely applied in various fields. However, due to constraints such as insufficient computing resources, it remains difficult to achieve high-speed structured light point cloud computation. To address this issue, this study proposes a memory-driven computational framework for accelerating point cloud computation. Specifically, the point cloud computation process is precomputed as much as possible and stored in memory in the form of parameters, thereby significantly reducing the computational load during actual point cloud computation. The framework is instantiated in two forms: a low-memory method that minimizes memory footprint at the expense of point cloud stability, and a high-memory method that preserves the nonlinear phase–distance relation via an extensive lookup table. Experimental evaluations demonstrate that the proposed methods achieve comparable accuracy to the conventional method while delivering substantial speedups, and data-format optimizations further reduce required bandwidth. This framework offers a generalizable paradigm for optimizing structured light pipelines, paving the way for enhanced real-time 3D sensing in embedded applications.

## 1. Introduction

3D vision technology enables the acquisition of the precise three-dimensional (3D) information of targets, bringing unprecedented efficiency and accuracy to the fields of measurement and inspection. It has become an indispensable technology in domains such as autonomous driving [1], virtual reality [2], geographic mapping [3], and intelligent manufacturing [4]. 3D vision technology encompasses various techniques, including stereo vision technology [5], time-of-flight (ToF) technology [6], photometric stereo technology [7], speckle structured light technology [8], and fringe structured light technology [9]. Among these 3D vision techniques, fringe structured light technology is widely applied in scenarios like intelligent welding [10], defect detection [11], intelligent processing [12], and intelligent assembly [13], owing to its advantages of high measurement accuracy, non-contact operation, and excellent robustness. However, its insufficient reconstruction speed restricts its application in many fields [14,15,16].

One way to enhance reconstruction speed is to improve hardware performance, thereby enabling faster fringe projection, image acquisition, and point cloud computation. In terms of boosting projection speed, binary defocus projection technology replaces 8-bit sinusoidal fringes with 1-bit binary fringes, increasing the fringe projection frequency to several thousand Hz [17,18,19]; the moving grating method uses a motor to drive the movement or rotation of a physical grating, allowing the projection of structured light fringes with a frequency of tens of thousands of Hz [20,21,22]; the LED array method leverages the high-speed switching capability of LED chips to achieve 1000 Hz fringe projection [23,24,25]; and the MEMS scanning method enables the projection of laser fringes at several thousand Hz by means of the rapid rotation of a MEMS scanning mirror [26].

For improving imaging speed, researchers have universally opted for high-speed cameras. Specifically, in the 3D reconstruction system developed by Chen et al., the camera employed achieves a frame rate of 1000 fps [27]; Feng et al. used the V611 high-speed camera with a frame rate of 20,000 fps to capture structured light images of dynamic scenes [28]; and in Wang et al.’s 3D reconstruction system, the camera utilized has a frame rate of 100,000 fps [29]. Regarding the acceleration of 3D reconstruction computational speed, researchers have utilized high-performance central processing units (CPUs) [30], graphics processing units (GPUs) [31], field-programmable gate arrays (FPGAs) [32,33,34], and application-specific integrated circuits (ASICs) [35] to enhance the system’s computational efficiency.

Another approach to improving reconstruction speed is to enhance the encoding efficiency of structured light. To this end, a series of structured light encoding methods have been proposed. Among these, spatial phase calculation methods enable the extraction of structured light phase information using only a single fringe pattern [36]. These methods include the Fourier transform method [37,38], windowed Fourier transform (WFT) method [39], and wavelet transform (WT) method [40]. Although these methods require a small number of fringe patterns, their global processing nature results in insufficient reconstruction accuracy when measuring objects with complex shapes—particularly the inability to obtain accurate phase information at object edges [41,42,43].

Temporal phase calculation methods utilize multiple phase-shifted fringe patterns to reconstruct the 3D shape of objects, enabling pixel-level measurement resolution. They are particularly suitable for 3D reconstruction of objects with complex shapes. However, due to the arctangent operation, phase information is confined to the interval (−π, π], thus requiring phase unwrapping algorithms to restore phase continuity [44,45]. Among phase unwrapping algorithms, the Gray code method requires fringe images for unwrapping; when combined with three images for phase shifting, the total number of images will exceed 10 [46]. To reduce the number of images required, the three-frequency three-step phase-shifting method computes wrapped phases using three-step phase shifting and only needs nine fringe images to calculate the absolute phase [47]. Thereafter, the two-frequency three-step phase-shifting method reduces the number of fringe images to six [48]. Liu et al. proposed a two-frequency pattern scheme that combines high-frequency sinusoidal components with unit-frequency components, further reducing the required images to five [49]. Additionally, Zuo et al. employed the Dual-Frequency Phase Shifting (DFPS) method, which only requires four fringe patterns to recover the absolute phase of the measured object [50,51]. Additionally, Zhang et al. introduced a lookup table to establish the relationship between wrapped phase and depth and leveraged speckle patterns to resolve depth ambiguities, thereby accelerating point cloud computation [52].

While the aforementioned methods enable systems to rapidly acquire the absolute phase of structured light fringes, calculating the 3D point cloud of the measured object from the absolute phase still consumes significant time. Methods for computing the 3D point cloud based on the absolute phase of structured light fringes include the classic phase–height model [53], linear phase–height model [54], linear inverse phase–height model [55], polynomial phase–height model, governing equation-based phase–height model [56], and triangular stereo model [57]. When using the aforementioned models to calculate the 3D point cloud, three key steps are required: firstly, complex distortion correction must be performed on the projector and camera lenses; secondly, except for the linear phase–height model (though it offers relatively faster computation speed, it exhibits larger errors compared to other models), nonlinear function operations or equation-solving operations are necessary to compute the depth data corresponding to each pixel of the camera; finally, the 3D coordinates of the point cloud corresponding to each pixel must be calculated based on the depth data and the calibrated internal parameters of the camera and projector [58]. The computational complexity of this process increases linearly with the number of camera pixels. This causes high-resolution structured light 3D reconstruction systems to consume substantial computational resources and time, thereby resulting in insufficient reconstruction speed.

The aforementioned issues are more pronounced in embedded structured light cameras. Embedded structured light cameras are 3D cameras designed and mass-produced based on structured light 3D reconstruction technology and embedded technology. They possess advantages such as small size, high precision, low cost, low power consumption, and strong robustness, and are widely used in scenarios including intelligent welding, defect detection, and intelligent assembly. Due to constraints imposed by factors like size, cost, and power consumption, it is difficult to integrate high-performance computing platforms into embedded structured light cameras. If the captured structured light images are uploaded to a host computer for processing, due to limitations such as transmission bandwidth, it is increasingly challenging to meet the on-site real-time performance requirements for 3D cameras. Furthermore, with the rapid development of humanoid robots, the demand for 3D vision devices featuring small size, high precision, high speed, low power consumption, low cost, and strong robustness has grown increasingly intense. Thus, determining how to rapidly compute the 3D point cloud coordinates of the measured object using limited computational resources in embedded structured light cameras has gradually become an urgent demand in this industry.

This paper proposes a unified memory-based acceleration framework for structured light 3D reconstruction. This framework formalizes memory-driven accelerations and provides a systematic way to analyze memory footprint, online computation, and reconstruction fidelity. This framework identifies common computational bottlenecks as ‘memorizable tasks’ and proposes a comprehensive approach to optimize them based on available memory resources, offering both low-memory and high-memory strategies. This novel abstraction allows for a more general, flexible, and scalable approach to acceleration, guiding the design of efficient embedded structured light systems.

## 2. Fundamentals of Structured Light 3D Reconstruction

### 2.1. Principles of Structured Light 3D Reconstruction

**Figure 1:**
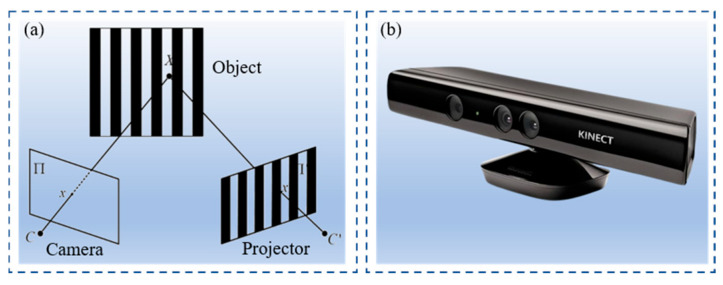
Structured light 3D reconstruction system: (**a**) principle schematic diagram; (**b**) actual product.

A structured light 3D reconstruction system is mainly composed of three components: a projector, a camera, and a computation module. During operation, the projector projects encoded structured light onto the measured object, forming a structured light pattern on its surface. Then, the camera captures structured light images of the measured object and transmits these images to the computation module. Finally, the computation module calculates the 3D point cloud of the measured object based on the captured structured light images and calibration parameters. The principle schematic diagram and the actual product are shown in Figure 1.

For any pixel in a structured light image, when the measured object undergoes displacement relative to the camera and projector, the structured light phase of this pixel changes accordingly. That is, different phase values correspond to different distances between the measured object and the structured light 3D reconstruction system. Thus, by pre-calibrating the correspondence between phase values and distances, the distance (i.e., the Z-coordinate of the spatial point) can be calculated based on the phase values in practical applications. Then, combined with the pre-calibrated X- and Y-coordinate parameters, the 3D point cloud of the measured object can be reconstructed.

When calculating point cloud using the structured light method, it is first necessary to perform distortion correction on the structured light images, followed by computing the Z-coordinate distance using epipolar geometry. These two computational processes are both complex and time-consuming. However, if the correspondence between different phase values and 3D point clouds is pre-established, then once the phase value of each pixel in the structured light image is obtained, the corresponding 3D spatial point can be retrieved directly from memory without additional computation—this will significantly enhance the system’s 3D reconstruction speed.

### 2.2. System Intrinsic Parameter Calibration

The intrinsic parameter calibration of the system mainly refers to the calibration of camera parameters, and the pinhole imaging model is typically adopted as the calibration model. In the pinhole imaging model, a spatial point $X_{W}$ is projected onto a point $x_{C}$ on the camera’s image plane via the optical center. Then, the coordinates $\left(X_{w},Y_{w},Z_{w},1\right)$ of the spatial point $X_{W}$ and the coordinates (u,v,1) of the image point $x_{C}$ are related by the following equation:

(1) $$s\left[\begin{array}{c} u\\ v\\ 1 \end{array}\right]=K\left[R|t\right]\left[\begin{array}{c} X_{w}\\ Y_{w}\\ Z_{w}\\ 1 \end{array}\right]=\left[\begin{array}{ccc} f_{u} & \gamma & u_{0}\\ 0 & f_{v} & v_{0}\\ 0 & 0 & 1 \end{array}\right]\left[R|t\right]\left[\begin{array}{c} X_{w}\\ Y_{w}\\ Z_{w}\\ 1 \end{array}\right]$$

where K is the intrinsic matrix of a camera; s is the scale factor; $f_{u}$ and $f_{v}$ denote the effective focal lengths along the u-axis and v-axis, respectively; γ represents the camera’s skew parameters; $\left(u_{0},v_{0}\right)$ are the principal point coordinates of the image plane; and R and t are the rotation matrix and translation vector between the camera coordinate system and the world coordinate system, respectively.

Equation (1) assumes that the camera’s imaging model is a standard linear model. However, due to constraints from manufacturing and assembly processes, there is a certain deviation between actual images and those under the pinhole imaging model—i.e., distortion exists. The main manifestations of image distortion are radial distortion and tangential distortion, which are usually expressed mathematically as:

(2) $$\left\{\begin{array}{c} \hat{x}=x\left(1+k_{1}r^{2}+k_{2}r^{4}+k_{3}r^{6}\right)+\left[2p_{1}xy+p_{2}\left(r^{2}+2x^{2}\right)\right]\\ \hat{y}=y\left(1+k_{1}r^{2}+k_{2}r^{4}+k_{3}r^{6}\right)+\left[2p_{2}xy+p_{1}\left(r^{2}+2y^{2}\right)\right]\\ r=\sqrt{x^{2}+y^{2}} \end{array}\right.$$

where $\left(x,y\right)$ and $\left(\hat{x},\hat{y}\right)$ denote the normalized coordinates of pixel $\left(u,v\right)$ on the image plane under ideal and actual conditions, respectively. $\left(k_{1},k_{2},k_{3}\right)$ are the radial distortion coefficients and $\left(p_{1},p_{2}\right)$ are the tangential distortion coefficients. Zhang Zhengyou’s calibration method can calibrate these camera parameters, offering high calibration accuracy and convenient operation [59].

## 3. Memory-Based Acceleration Framework of Point Cloud Computation

### 3.1. Two-Parameter Phase–Distance Model

**Figure 2:**
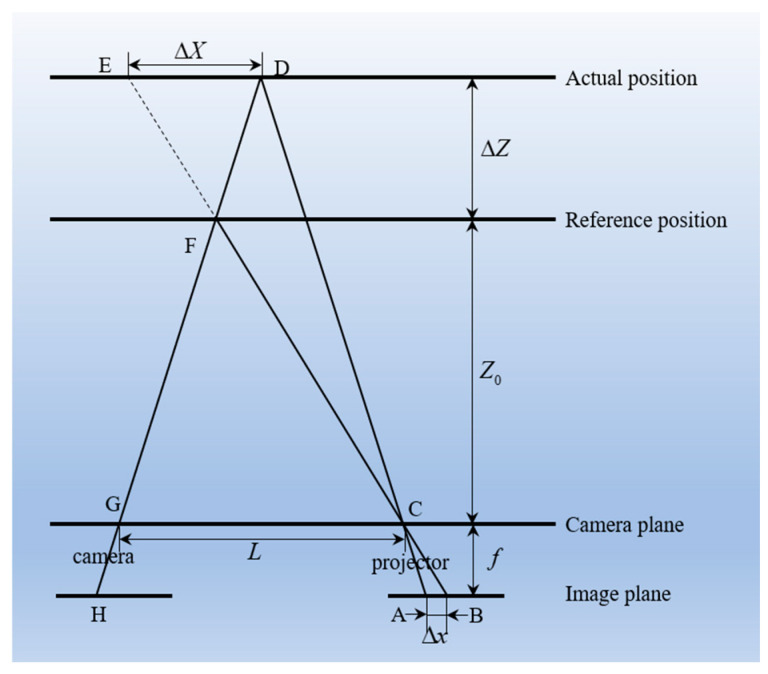
Schematic diagram of the phase–distance relationship in the structured light system.

Figure 2 shows a schematic diagram of the phase–distance relationship in the structured light system. The reference position and actual position correspond to two distinct positions of the object. The optical center **G** of the camera and the optical center **C** of the projector lie on the camera plane, with the distance between the two optical centers being L. Both the camera and the projector form images on the image plane. The focal length of the projector is f; the axial distance from the reference position to the camera plane is $Z_{0}$; and the axial distance between the actual position and the reference position is ΔZ.

For any pixel **H** on the camera image plane, when the object is at the reference position, the fringes projected from point **B** on the projector image plane reach pixel **H** of the camera after being reflected by point **F** on the object. When the object is at the actual position, the fringes projected from point **A** on the projector image plane reach pixel **H** of the camera after being reflected by point **D** on the object. The distance between points **A** and **B** on the projector image plane along the fringe scanning direction is Δx.

From the similarity of triangles ABC and DEC (ΔABC~ΔDEC), it can be derived that:

(3) $$\frac{\Delta x}{f}=\frac{\Delta X}{Z_{0}+\Delta Z}$$

From the similarity of triangles CGF and EDF (ΔCGF~ΔEDF), it can be derived that:

(4) $$\frac{L}{\Delta X}=\frac{Z_{0}}{\Delta Z}$$

Furthermore, on the image plane of the projector,

(5) Δx=kΔα

where Δα denotes the structured light phase difference between points **A** and **B** and k represents the ratio of distance to phase along the fringe scanning direction on the projector image plane.

By combining Equations (3)–(5), the following can be derived:

(6) $$\Delta Z=\frac{kZ_{0}^{2}\Delta \alpha }{Lf-kZ_{0}\Delta \alpha }$$

In Equation (6), $Z_{0}$, L, f, and k are all constant values. Let:

(7) $$\left\{\begin{array}{c} a_{u,v}=\frac{Lf}{kZ_{0}^{2}}\\ b_{u,v}=-\frac{1}{Z_{0}} \end{array}\right.$$

Then Equation (6) can be simplified to:

(8) $$\Delta Z=\frac{\Delta \alpha }{a_{u,v}+b_{u,v}\Delta \alpha }$$

Thus, as long as the $a_{u,v}$ and $b_{u,v}$ values for each pixel are determined, the relationship between phase and distance can be established.

In the model above, $a_{u,v}$ represents the rate of change in depth with respect to phase at the pixel (u,v), which is primarily influenced by the baseline (distance between camera and projector), the focal lengths of the camera and projector, and the relative projection angle at that specific pixel; $b_{u,v}$ acts as an offset or intercept, effectively shifting the entire phase–distance curve along the depth axis, which is largely determined by the absolute position of the reference plane during calibration. A larger absolute value of $a_{u,v}$ implies that a small error in the measured phase will result in a proportionally larger error in the estimated depth. This makes pixels with large $a_{u,v}$ values more susceptible to phase noise or quantization errors.

During the calibration process (least-squares fitting for $a_{u,v}$ and $b_{u,v}$), the residual error for each pixel provides a critical indicator of how well the linear model fits the actual measured phase-depth data. A high residual value for a pixel suggests that the linear approximation is poor for that pixel or over the calibrated depth range, indicating that the low-memory method might be unreliable for that specific region.

**Figure 3:**
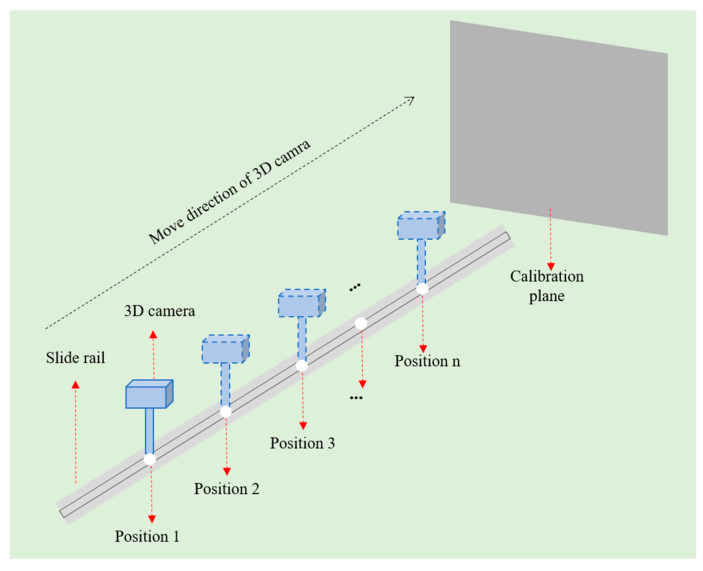
Schematic diagram of the calibration of phase and depth values.

To determine $a_{u,v}$ and $b_{u,v}$, the measurement range d of the structured light 3D reconstruction system can be equally divided into n segments. Starting from the initial position, the object is moved by a step distance of d/n each time. Subsequently, structured light fringes are projected and captured, and the structured light phase values of each pixel in the images at each position are calculated, as shown in Figure 3. Then, based on the moved distances and structured light phase values, the least squares method is used to calculate the values of $a_{u,v}$ and $b_{u,v}$ for each pixel separately. By taking the reciprocal of $b_{u,v}$, the value of $Z_{0}$ can be calculated.

It should be noted that the per-pixel two-parameter phase–distance model in Equation (8) is derived under the following explicit assumptions: (i) a calibrated pinhole projection for both camera and projector with distortions accounted for by the intrinsic calibration; (ii) higher-order distortion terms (beyond first-order radial and tangential distortion) are negligible within the calibrated working volume; (iii) the measured phase is obtained from a robust demodulation pipeline and treated as a noisy but unbiased quantity; and (iv) $a_{u,v}$ and $b_{u,v}$ are treated as locally constant over the depth range used for calibration.

When fitting the values of $a_{u,v}$ and $b_{u,v}$ for each pixel,

(9) $$a_{u,v}=\frac{Lf}{k}b_{u,v}^{2}$$

Let:

(10) $$m=\frac{Lf}{k}$$

Then

(11) $$a_{u,v}=mb_{u,v}^{2}$$

In the image sensor, the values of m are the same for each pixel, while the values of $b_{u,v}$ are generally different.

### 3.2. Low-Memory Point Cloud Computation Method

From the *n* + 1 calibration positions, select any one position as the reference position. The phase of each pixel at this position is the reference phase $\alpha_{u,v,0}$, and the axial distance of each pixel at this position is $Z_{u,v,0}$.

During 3D reconstruction, after calculating the absolute phase values $\alpha_{u,v}$ of each pixel, the Z-directional distance of the spatial point corresponding to each pixel can be determined as follows:

(12) $$\begin{array}{l} Z_{u,v}=Z_{u,v,0}+\Delta Z_{u,v}\\ \;\;\;\;\;=Z_{u,v,0}+\frac{\Delta \alpha_{u,v}}{a_{u,v}+b_{u,v}\Delta \alpha_{u,v}}\\ \;\;\;\;\;=Z_{u,v,0}+\frac{\alpha_{u,v}-\alpha_{u,v,0}}{a_{u,v}+b_{u,v}(\alpha_{u,v}-\alpha_{u,v,0})} \end{array}$$

If the camera coordinate system is adopted as the world coordinate system, then the rotation matrix R is an identity matrix and the translation vector t is a zero vector.

(13) $$\left\{\begin{array}{c} R=\left[\begin{array}{ccc} 1 & 0 & 0\\ 0 & 1 & 0\\ 0 & 0 & 1 \end{array}\right]\\ t={\left[\begin{array}{ccc} 0 & 0 & 0 \end{array}\right]}^{T} \end{array}\right.$$

According to Section 3.1, $Z_{w}=Z_{u,v}$, and substituting this into Equation (1) yields:

(14) $$\begin{array}{c} X_{w}=\frac{{\tilde{u}}_{u,v}Z_{u,v}}{f_{x}}\\ Y_{w}=\frac{{\tilde{v}}_{u,v}Z_{u,v}}{f_{y}} \end{array}$$

where $\left({\tilde{u}}_{u,v},{\tilde{v}}_{u,v}\right)$ denotes the distortion-corrected pixel coordinates of each pixel point. Let:

(15) $$\left\{\begin{array}{c} ratio\_u_{u,v}=\frac{{\tilde{u}}_{u,v}}{f_{x}}\\ ratio\_v_{u,v}=\frac{{\tilde{v}}_{u,v}}{f_{y}} \end{array}\right.$$

Then the 3D coordinates of the spatial point cloud corresponding to each pixel point can be obtained:

(16) $$\left\{\begin{array}{c} X_{w}=ratio\_u_{u,v}*Z_{u,v}\\ Y_{w}=ratio\_v_{u,v}*Z_{u,v}\\ Z_{w}=Z_{u,v} \end{array}\right.$$

Since each calculation requires camera distortion correction and the calculation of $ratio\_u_{u,v}$ and $ratio\_v_{u,v}$ using calibrated internal parameters, if $ratio\_u_{u,v}$ and $ratio\_v_{u,v}$ for all pixels are precomputed and stored in the system memory, then during the 3D reconstruction process of the system, only calculations using Equations (12) and (16) are required to achieve 3D reconstruction of the measured object.

### 3.3. High-Memory Point Cloud Computation Method

Due to the projection errors of the projector and the defocus effect of the lens, the phase-shifted structured light fringes captured by the camera are not standard sinusoidal fringes. This results in fluctuations in the calculated absolute phase, thereby increasing the 3D reconstruction error of the system. In other words, the relationship between the calculated phase and the moving distance does not strictly follow Equation (12); instead, there exists a certain degree of error. To more accurately determine the relationship between the calculated phase and the moving distance, this paper employs a large memory space to establish a detailed lookup table of absolute phase and moving distance for implementation.

To achieve this, when the measurement range of the system is d and the desired measurement accuracy is ε, the number of moving positions n is given by:

(17) $$n=\frac{d}{\varepsilon }$$

Within the measurement range of the system, starting from the minimum measurement distance of the camera, we move n times with a step size of ε; this results in *n* + 1 positions, at which point the correspondence table between the absolute phase of each pixel and the Z-directional distance is measured.

To facilitate lookup, the phase difference between the first position and the nth position is divided into n equal parts. The phase value of pixel point $\left(u,v\right)$ at the m-th phase position is given by:

(18) $$\alpha_{u,v,m}=\alpha_{u,v,0}+m\delta_{u,v},\;\;\;m=0,1,2\;\ldots \;n$$

where $\alpha_{u,v,0}$ denotes the phase of the pixel point at the first position and $\delta_{u,v}$ denotes the phase difference between two adjacent phase positions:

(19) $$\delta_{u,v}=\frac{\alpha_{u,v,n}-\alpha_{u,v,0}}{n}$$

From the established correspondence table between phase values and Z-directional distances, we select the phase value with the smallest difference from the phase $\alpha_{u,v,m}$ and perform interpolation to obtain the distance corresponding to the phase $\alpha_{u,v,m}$. Then, we use Equation (19) to calculate the 3D coordinates of the spatial point corresponding to the phase $\alpha_{u,v,m}$. Repeating the above steps for the *n* + 1 phase positions of all pixel points yields the correspondence between phase values and spatial point coordinates at the *n* + 1 phase positions for each pixel point.

The above-mentioned spatial point coordinates are of float type or double type. In embedded platforms, float-type data typically occupies 4 bytes, while double-type data usually occupies 8 bytes. For value n, 255 ≤ n ≤ 65,535; thus, this study uses the short int type to store spatial point Z-coordinates. The short-int-type Z-coordinates of spatial point $\left(u,v\right)$ at the m-th phase position are given by:

(20) $$Z_{u,v,m}^{\prime }=\left\lfloor \frac{1}{\varepsilon }Z_{u,v,m}\right\rfloor$$

where$\left(X_{u,v,m},Y_{u,v,m},Z_{u,v,m}\right)$ denotes the float-type or double-type coordinate values of spatial point $\left(u,v\right)$ at the m-th phase position, calculated in Section 3.2; ε denotes the minimum measurement accuracy of the system; ⌊ ⌋ and denotes the rounding operator.

This paper uses an array to store the spatial point Z-coordinates of each pixel point at n phase positions. The element in the array is denoted as $d_{u,v,k}$.

(21) $$d_{u,v,k}=Z_{u,v,m}^{\prime }$$

Thus, the lookup table of spatial point Z-coordinates for each pixel point at *n* + 1 phase positions is established. Furthermore, to facilitate lookup, it is necessary to record the phase value at the initial position of each pixel point $\alpha_{u,v,0}$ and the phase step size $\delta_{u,v}$ in the system’s memory.

During 3D reconstruction, the phase value $\alpha_{u,v}$ of each pixel point is calculated; the position index corresponding to pixel point $\left(u,v\right)$ is given by:

(22) $$m^{\prime }=\left\lfloor \frac{\alpha_{u,v}-\alpha_{u,v,0}}{\delta_{u,v}}\right\rfloor$$

The short-int-type 3D coordinates of the spatial point corresponding to pixel point $\left(u,v\right)$ are given by:

(23) $$\left[\begin{array}{c} X_{W}^{\prime }\\ Y_{W}^{\prime }\\ Z_{W}^{\prime } \end{array}\right]=d_{u,v,m^{\prime }}\left[\begin{array}{c} ratio\_u_{u,v}\\ ratio\_v_{u,v}\\ 1 \end{array}\right]$$

The short-int-type 3D coordinates of each pixel point are calculated and uploaded to the host computer. Using Equation (24), the float-type 3D coordinates of the spatial point corresponding to each pixel point are decoded in the host computer; this not only reduces the storage space of the lookup table, but also decreases the data volume of data transmission.

(24) $$\left[\begin{array}{c} X_{W}\\ Y_{W}\\ Z_{W} \end{array}\right]=\varepsilon \left[\begin{array}{c} X_{W}^{\prime }\\ Y_{W}^{\prime }\\ Z_{W}^{\prime } \end{array}\right]$$

In practical structured light systems, phase maps often contain outliers due to low reflectivity, occlusions, or specular surfaces. To suppress these outliers, during calibration we estimate, for each pixel, the minimum absolute phase $\alpha_{\min}$ and the maximum absolute phase $\alpha_{\max}$ within the system’s measurement range. If the computed absolute phase lies outside this range, the corresponding depth value is set to zero, and no further computations or lookup table operations are performed for that pixel.

## 4. Experiment

**Figure 4:**
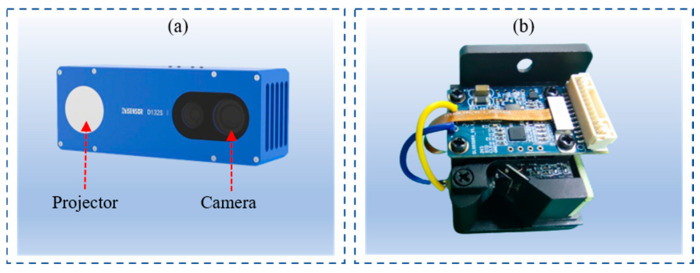
Experimental equipment: (**a**) structured light system; (**b**) projector.

To evaluate the actual performance of the proposed methods in this study, experiments are conducted using the D132S structured light camera from Xi’an Zhiwei Company (Xi’an, China). As shown in Figure 4a, the D132S camera consists of one structured light projector and one monochrome camera. The structured light projector adopts the P1130 MEMS uniaxial scanning module, which can project Gray code fringes and phase-shifted code fringes, featuring advantages such as high resolution, high reliability, low cost, small size, and easy integration. The monochrome camera has a resolution of 1280 × 1024 and can capture structured light grayscale images in RAW format. In this study, the aforementioned camera and projector are used to form a structured light 3D reconstruction system, with the RK3399 core board employed for data computation. During 3D reconstruction, seven-level Gray code and the four-step phase shifting method are adopted for encoding the structured light fringes.

### 4.1. Experiments on the Low-Memory Method

**Figure 5:**
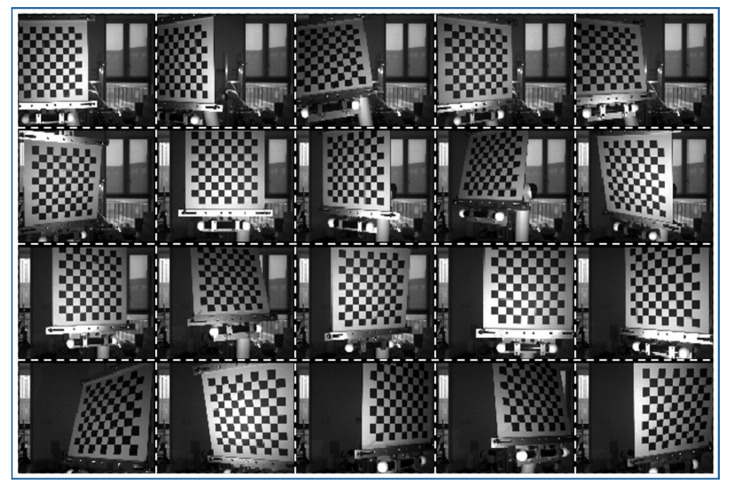
Chessboard pose images for calibrating camera’s intrinsic parameters.

This study employs the Zhang Zhengyou calibration method to calibrate the camera’s internal parameters. During calibration, 20 structured light images with different poses are captured, as shown in Figure 5, and the camera’s internal parameters are computed using OpenCV 3.4.1 software, with the computed results given by:

(25) $$\left\{\begin{array}{c} \left[\begin{array}{ccc} f_{u} & \gamma & u_{0}\\ 0 & f_{v} & v_{0}\\ 0 & 0 & 1 \end{array}\right]=\left[\begin{array}{ccc} 1.4328957\times 10^{3} & 0 & \;6.3751170\times 10^{2}\\ 0 & 1.4326590\times 10^{3} & 5.2187200\times 10^{2}\\ 0 & 0 & 1 \end{array}\right]\\ \left[\begin{array}{c} k_{1}\\ k_{2}\\ k_{3}\\ p_{1}\\ p_{2} \end{array}\right]=\left[\begin{array}{c} -1.2009005\times 10^{-1}\\ \;1.1928703\times 10^{-1}\\ 0\\ 9.6197371\times 10^{-5}\\ -1.4896083\times 10^{-4} \end{array}\right] \end{array}\right.$$

According to Equation (15), after the coordinates of each pixel are calibrated, the ratio of the X-coordinate to the Z-coordinate $ratioX_{u,v}$ and the ratio of the Y-coordinate to the Z-coordinate $ratioY_{u,v}$ of the point clouds corresponding to each pixel are computed respectively. These ratios are then stored in the memory of the structured light camera for use in 3D reconstruction.

**Figure 6:**
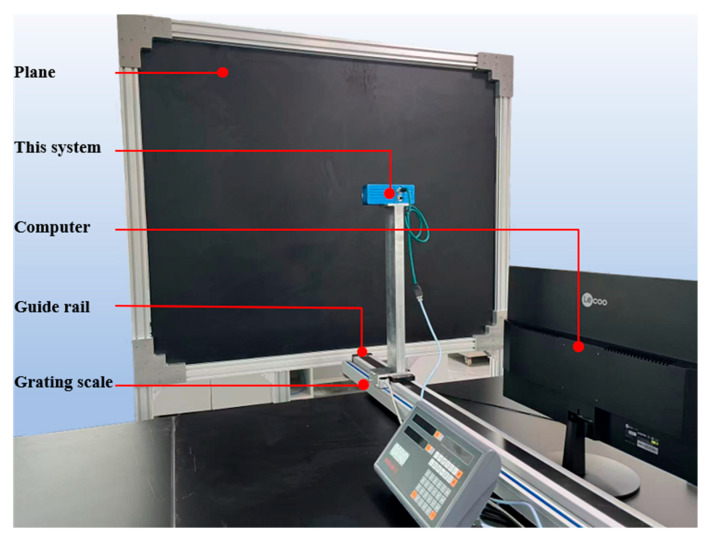
System calibration environment.

As shown in Figure 6, the camera is fixed on a guide rail via a mechanical bracket, with the guide rail perpendicular to the calibration plane; the distance between the structured light system and the calibration plane is adjusted by moving the guide rail. The camera has a working range of 400 mm to 600 mm. Starting from 400 mm, the camera is moved by 2 mm each time, and the moving distance of the camera is recorded based on the readings of the grating scale. Meanwhile, a set of structured light fringe images is captured, and the absolute phase value of each pixel is computed. According to the differences between the absolute phase values at each position and those at the initial position, as well as the moving distances, the a-value $a_{u,v}$ and b-value $b_{u,v}$ for each pixel are fitted respectively according to Equation (8). These a-values and b-values are then stored in the memory of the structured light camera.

**Figure 7:**
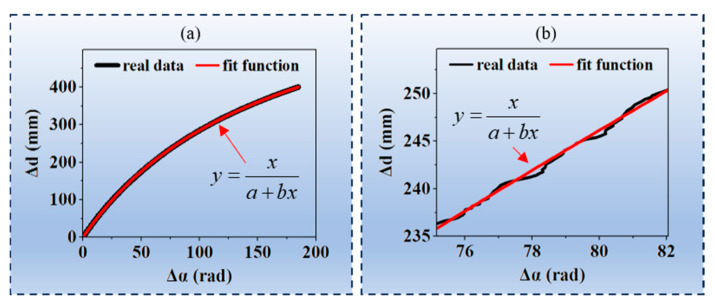
Actual data and fitted curve of the phase difference and distance difference for the pixels (500, 600): (**a**) full-range data; (**b**) data in the 75 rad–82 rad range.

During testing, it was found that the point cloud reconstructed by this method exhibited significant fluctuations. The study of its fluctuation errors revealed that, due to the projection errors of the projector and the defocus effect of the lens, the relationship between the absolute phase and the moving distance did not strictly follow Equation (8); instead, it exhibited a certain degree of fluctuation relative to the curve of Equation (8), as shown in Figure 7b, thereby leading to calculation errors in the point cloud.

It should be noted that this fluctuation error is not intrinsic to the proposed two-parameter phase–distance models but is a generic characteristic of all phase-to-depth models in structured light systems. The relationship between the precise absolute phase and displacement strictly follows Formula (8) in theory. However, in practical applications, when the period of the phase-shifted structured light patterns is very small, the actual projected fringes are often not perfectly sinusoidal. This deviation from ideal sinusoidal patterns introduces errors in the absolute phase calculation. The poorer the sinusoidal quality of the projected fringes, the greater the error in the calculated absolute phase. Consequently, the curve representing the absolute phase with these calculation errors, plotted against displacement, does not strictly adhere to Formula (8).

When the projector’s projection error lies within the system’s allowable tolerance, the effect can be neglected. If the projection error becomes large, compensation should be applied or an alternative modeling approach should be used for computation. For applications where computational resources are highly constrained and a small compromise in accuracy is acceptable, the low-memory method offers a superior trade-off by drastically reducing online computation. Its suitability should be evaluated by empirical calibration and by checking the residual fitting errors against the application’s accuracy requirements. If these residuals are too high, or if the depth range is too wide and nonlinear, the high-memory method becomes a more suitable alternative.

### 4.2. Experiments on the High-Memory Method

To address the aforementioned point cloud fluctuation issue, this paper further explored a high-memory 3D reconstruction method. The system employed in this study has a working range of 400 mm to 600 mm and a target measurement accuracy of 0.2 mm. By moving the guide rail 1000 times in accordance with the method described in Section 3.3, the correspondence table between the absolute phase of each pixel and the 3D point cloud was established, as well as the phase value table at the initial position for each pixel and the phase step size table. During operation, the table position of each pixel could be located in accordance with Equation (12), thereby achieving 3D reconstruction of the measured object.

**Figure 8:**
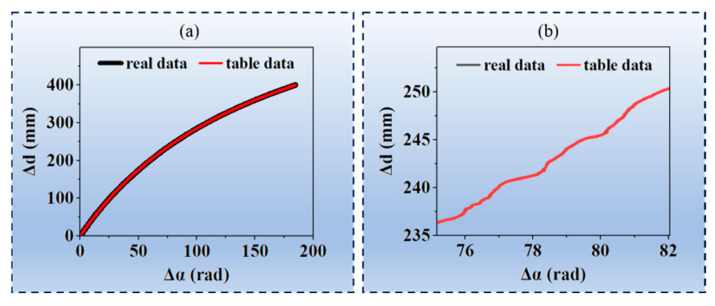
Actual data and table data of the phase difference and distance difference for the pixels (500, 600): (**a**) full-range data; (**b**) data in the 75 rad–82 rad range.

For this method, the correspondence table between the absolute phase and the 3D point cloud is established based on true values. The table data and true data are as shown in Figure 8. It can be observed from Figure 8b that the table data and the true data nearly coincide.

### 4.3. Comparison Experiment

To validate the performance of the proposed methods, this paper compared them with the light-plane method. This method reconstructs 3D points by projecting the stripe boundary lines of the structured light pattern and then back-projecting the corresponding image pixels from the camera center. The intersection of these back-projected pixels and the observed stripe boundary lines (light planes) on the object surface yields the 3D reconstruction points. This method is widely adopted in industrial applications for its fast reconstruction speed, high accuracy, and strong robustness. The light-plane method uses the identical structured light patterns and the same hardware platform as those employed by the proposed two methods. The three methods are further compared from three aspects: computation time, planar RMS accuracy, and standard sphere accuracy. To ensure statistical reliability, the experiment is repeated 10 times, and the mean values are calculated to determine reconstruction time and accuracy for each experiment. The experimental setups for the three methods are detailed in Table 1.

In Table 1, $l_{1,n}$,$l_{2,n}$,$l_{3,n}$, and $l_{4,n}$ denote the parameters of the nth light-plane equation.

(26) $$l_{1,n}X_{W}+l_{2,n}Y_{W}+l_{3,n}Z_{W}+l_{4,n}=0$$

#### 4.3.1. Comparison of Computation Time

This study tested the computation time of the three methods using both the CPU and GPU of the RK3399 computing platform, with the test results presented in Table 2.

As shown in Table 2, owing to the elimination of numerous computation steps, the computation time of the two methods proposed in this study has achieved a reduction by an order of magnitude compared with that of the light-plane method. Although the high-memory method involves fewer computation steps than the low-memory method, due to its larger memory footprint, its addressing time is longer than that of the low-memory method. Consequently, the computation times of the two methods are roughly comparable. Additionally, in the high-memory method, since the point cloud data type is changed from float to unsigned short, the data storage space is reduced by half, and thus the transmission time is also significantly reduced.

#### 4.3.2. Comparison of Planar RMS Accuracy

**Figure 9:**
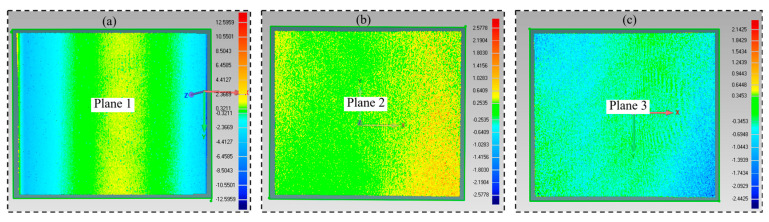
Fitted point cloud: (**a**) fitted point cloud of the low-memory method; (**b**) fitted point cloud of the high-memory method; (**c**) fitted point cloud of the light-plane method.

During the measurement of planar RMS accuracy, the structured light camera is moved to a non-calibrated distance position, a set of structured light images is captured, and the absolute phase of each pixel is then computed. Based on the absolute phase of each pixel, the point cloud of this calibration plane is computed using the three methods respectively. The acquired point cloud is further fitted using Geomagic software, with the fitting results shown in Figure 9. The planar RMS errors of the three methods are shown in Table 3.

As can be observed from Figure 9 and Table 3, since the similarity of the table data to the true data in the high-memory method is better than that of the fitted curve to the true data in the low-memory method, the RMS accuracy of the high-memory method is significantly superior to that of the low-memory method. However, due to the interpolation errors during the lookup table establishment process in the high-memory method and the rounding errors caused by the data type conversion from float to unsigned int, the RMS accuracy of the high-memory method is inferior to that of the light-plane method. These quantization processes inherently introduce a certain degree of computational error. If this error falls within the system’s required accuracy range, it can be neglected. However, when this error becomes substantial and severely impacts the system’s precision, mitigation strategies such as adopting a finer quantization step size or employing methods like interpolation and spatial filtering can be utilized to suppress noise. Nevertheless, these processes inevitably increase memory consumption and computational complexity, consequently increasing the 3D reconstruction time of the system.

#### 4.3.3. Comparison of Standard Sphere Accuracy

**Figure 10:**
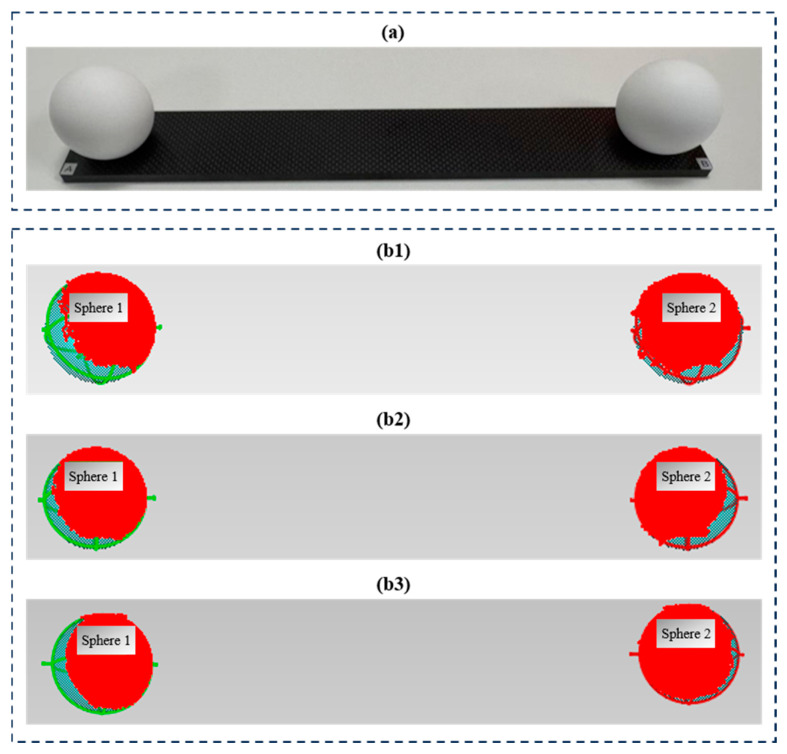
Ceramic standard spheres reconstructed by different methods: (**a**) two standard dumbbell-shaped ceramic spheres, (**b1**) point cloud fitting results of the low-memory method, (**b2**) point cloud fitting results of the high-memory method, (**b3**) point cloud fitting results of the light-plane method.

To further test the measurement accuracy of the three methods, two standard dumbbell-shaped ceramic spheres were selected as the test targets, as shown in Figure 10a. The diameters of the two ceramic standard spheres were 50.8019 mm and 50.8017 mm respectively, and they were mounted on a carbon fiber plate, with a center-to-center distance of 299.8513 mm between the two spheres. The reconstructed point clouds of the three methods were fitted using Geomagic software, with the fitting results as shown in Figure 10 and the computed point cloud errors presented in Table 4.

As shown in Figure 10 and Table 4, although the low-memory method exhibits significant point cloud fluctuations, the standard sphere accuracy of the three methods shows no significant difference, thereby validating the feasibility of the proposed methods in this study.

## 5. Discussion

### 5.1. Trade-Off Between Accuracy and Memory in High-Memory Methods

In the high-memory approach described herein, memory consumption depends on the camera resolution, the number of calibration positions, and the data storage type. When the camera resolution is W × H, the number of calibration locations is n+1 and the data storage type is data_type; the memory footprint is given by Equation (27).

(27) $$Memory=W\times H\times ((n+1)\times sizeof\left(data\_type\right)+16)$$

In this equation, the value 16 denotes the $\alpha_{i,j,0}$, $\alpha_{\min}$, $\alpha_{\max}$, and $\delta_{i,j}$ that need to be stored. In this paper, the camera resolution is 1280 × 1024, the number of calibration locations is 1001, and the data storage type is short (16-bit integer), so the total memory consumption is determined by:

(28)  Memory =1280×1024×((1001+1)×2(bytes)+16)≈2.5GB

As shown in Equation (17), the number of calibration locations is determined by both the measurement range and the required measurement accuracy. For a fixed system setup, the measurement range is consequently fixed. Therefore, the relationship between measurement accuracy and memory footprint is as follows:

(29) $$Memory\;=W\times H\times ((\frac{d}{\varepsilon }+1)\times sizeof\left(data\_type\right)+16)$$

**Figure 11:**
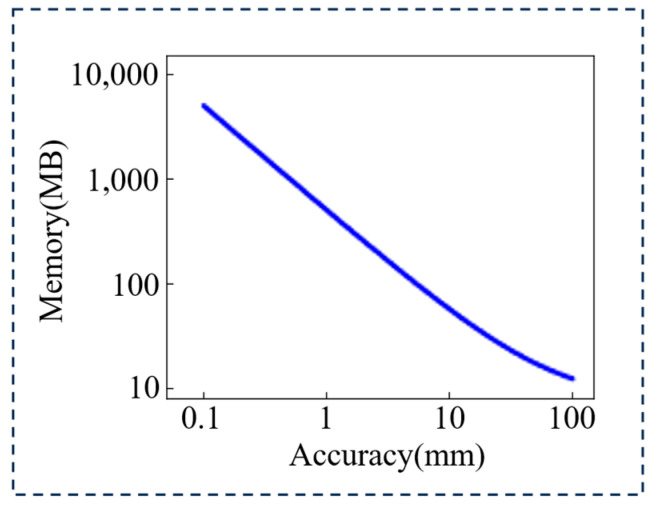
The relationship between measurement accuracy and memory footprint in the high-memory method.

In this study, the relationship between memory footprint and measurement accuracy is illustrated in Figure 11.

As illustrated in Figure 11, an increase in measurement precision for the high-memory method leads to a progressively larger system memory footprint. This phenomenon arises because enhanced calibration precision is primarily achieved by decreasing the calibration step size. Such a reduction, however, invariably leads to a proportional increase in the number of calibration points, consequently expanding the memory footprint.

However, this improvement in calibration precision is not limitless. This is primarily due to two factors. Firstly, the calibration equipment possesses inherent limitations in its minimum movement step and positioning accuracy; consequently, reducing the calibration step size below a certain threshold will not yield further improvements in measurement precision. Secondly, owing to factors such as assembly and manufacturing tolerances, the structured light 3D reconstruction system itself has an intrinsic limit to its reconstruction accuracy, which the calibrated system cannot surpass.

In addition, it is worth noting that constantly expanding the memory may not necessarily boost the reconstruction speed because on such embedded systems, memory bandwidth and cache performance (including L1, L2, and potentially L3 caches) can become significant bottlenecks, potentially negating the benefits of reduced computation. Accessing large lookup tables, particularly when the access pattern is non-sequential or ‘random’, can lead to frequent cache misses. A cache miss results in a delay as data must be fetched from slower main memory (DDR), thereby increasing memory latency and reducing overall processing speed. If the access pattern is highly random and spans across large portions of memory, it can lead to ‘cache thrashing’, where useful data is constantly evicted from the cache to make room for new data that is soon also evicted, further diminishing performance.

During the real-time reconstruction process, the system typically processes image frames in a pixel-by-pixel or scanline-by-scanline manner. This sequential access pattern for the coordinates inherently possesses spatial locality. As the processing moves from one pixel to an adjacent one, it is highly probable that the data required for the neighboring pixel (including its 3D coordinates or related parameters) will reside in the same or nearby cache lines. However, when pixels are located at object edges or in regions with complex geometries, the phase values of adjacent pixels can vary significantly, thereby increasing the randomness of memory access and consequently affecting the system’s reconstruction speed. Therefore, our future work would explore data structures or compression techniques that further enhance spatial or temporal locality during access, e.g., by organizing the memory in a hierarchical manner, to potentially reduce cache thrashing.

### 5.2. Comparison with Existing Acceleration Methodologies

This work introduces a memory-based acceleration framework for structured light 3D reconstruction. It is not claimed as a universal algorithmic breakthrough for all structured light pipelines; instead, it provides a unified theoretical abstraction and practical instantiations that systematically analyze memory–accuracy trade-offs.

The principle of trading computation for memory has been applied in some fields, including structured light 3D reconstruction. Several existing approaches implicitly or explicitly leverage this concept to enhance performance. It is crucial to distinguish our proposed memory-based acceleration framework from these related methodologies to clarify its unique contribution.

Traditional calibration-based lookup tables (LUTs), such as phase–height models or calibration tables for depth recovery, represent a common form of memory-for-computation trade-off. These methods precompute the relationship between measured optical features (e.g., phase) and 3D spatial coordinates for a specific camera-projector setup and working volume. Existing lookup tables are typically small and primarily used for phase correction.

Similarly, FPGA/ASIC implementations often incorporate lookup tables as low-level hardware components. These hardware LUTs are fundamental building blocks designed to execute specific combinatorial logic rapidly or to store small, frequently accessed data sets, directly contributing to hardware-accelerated processing. The optimization focus here is primarily at the hardware description level, aiming for minimal gate delays and efficient resource utilization within the physical circuit.

In contrast, our proposed memory-based acceleration framework operates at a higher algorithmic and theoretical abstraction level. It is not a specific precomputation table, nor is it a hardware-centric optimization technique. Instead, it offers a systematic framework for analyzing the entire structured light reconstruction pipeline, identifying diverse computational stages as ‘memorizable tasks’—not limited to geometric mapping. This framework provides a principled methodology to:

(1) Identify Memorizable Tasks: Systematically pinpoint computational operations that can be transformed into memory lookups or caching mechanisms;
(2) Define Memory Budgeting: Guide the process of establishing and managing memory resources effectively;
(3) Formulate Memory-Computation Strategies: Develop flexible acceleration strategies (e.g., low-memory and high-memory) based on the available memory budget, real-time constraints, and desired reconstruction quality.

This holistic approach moves beyond merely precomputing specific geometric relations or accelerating isolated hardware functions. It provides a generic design paradigm for making informed decisions about how to best exploit memory to reduce computational load across a broad spectrum of structured light algorithms and their various processing stages.

### 5.3. Main Contributions of This Work

After over a decade of unremitting efforts by researchers, fringe structured light 3D reconstruction technology has made remarkable progress both in terms of speed and accuracy. Specifically, its reconstruction speed can reach an impressive tens to hundreds of kHz. However, in industrial settings, structured light cameras with a reconstruction speed of 10 Hz are still extremely rare. One important reason for this is that high-speed structured light 3D reconstruction technology typically requires high-performance data processing equipment, whereas structured light cameras used in industry usually adopt embedded platforms. Due to insufficient computing performance, embedded platforms often struggle to cope with the processing demands of high-speed structured light 3D reconstruction algorithms.

To address this issue, this study proposes a framework to accelerate point cloud computation for embedded structured light cameras using memory. Specifically, the point cloud computation process is precomputed as much as possible and stored in memory in the form of parameters, thereby significantly reducing the computational load during actual point cloud computation. To the best of our knowledge, this is the first time this framework has been explicitly proposed to specifically improve the speed of embedded structured light cameras in this manner. The framework is instantiated in two forms: a low-memory method and a high-memory method. By comparing with the light-plane method, it is demonstrated that the proposed two methods significantly improve the point cloud reconstruction speed while achieving comparable accuracy performance. The main contributions of this study are as follows:

(1) **Memory method to accelerate point cloud computation**: The point cloud computation process is precomputed as much as possible and stored in memory in the form of parameters, thereby significantly reducing the computational load during actual point cloud computation. The framework does not rely on any particular approach to computing the absolute phase, enabling potential transfer to other structured light modalities (e.g., binary defocus projection and LED array projection) in 3D reconstruction systems.
(2) **Low-memory point cloud computation method**: The principle of the two-parameter point cloud reconstruction method, as well as the mathematical relationship between parameter $a_{u,v}$ and parameter $b_{u,v}$, has been geometrically derived; the calibration process for the two parameters in this method under constraint conditions has been proposed; by pre-calibrating the lens and storing the calibration results in memory in the form of parameters, the computational load of point clouds is significantly reduced.
(3) **High-memory point cloud computation method**: The calibration process and point cloud computation process for the high-memory point cloud computation method are proposed, which maintain the nonlinear relationship between the system’s absolute phase and moving distance to the greatest extent possible.
(4) **Memory-saving method via data type conversion**: By converting the data type from float to unsigned short, the storage space for calibration parameters and the bandwidth for point cloud transmission are significantly reduced.

Despite these advantages, the methods proposed still have certain limitations. In the low-memory point cloud computation method, real data fluctuates along the function curve, thereby introducing point cloud reconstruction errors. The specific mathematical relationship of this fluctuation requires further investigation. In the high-memory point cloud computation method, thousands of guide rail movements are required, the calibration process takes 2 h, and the calibration parameters occupy significant storage space. In the future, in-depth research will be conducted on these two issues to achieve simultaneous improvements in both calibration speed and reconstruction speed.

## 6. Conclusions

Due to insufficient computing resources, embedded structured light cameras typically exhibit low 3D reconstruction speeds. To address this issue, this study proposes a memory-based acceleration framework for structured light 3D reconstruction and demonstrates two concrete instantiations: a low-memory method and a high-memory method. The framework provides a unified abstraction to analyze memory usage, online computation, and reconstruction fidelity. Initial embedded-platform experiments show meaningful speedups with controllable accuracy implications. This theoretical framework provides a robust and widely applicable paradigm for enhancing the performance of structured light reconstruction algorithms, offering a systematic way to analyze and design memory-centric accelerations across various algorithmic components. Future work will explore the application of this framework to learning-based structured light methods and its integration with advanced hardware architectures to further push the boundaries of real-time 3D sensing.

## Figures and Tables

**Table 1:** Experimental setups for the three methods.

	Low-Memory	High-Memory	Light-Plane
Hardware	Computing Platform: RK3399 Camera Resolution:1280 × 1024 Projector: P1130 MEMS projector Memory: 4 GB		
Software	Operating System: Ubuntu22.04 Programming Language: C/OpenCL		
Calibration Parameters	$f_{u}$ $,f_{v}$ γ $,u_{0}$ $,v_{0}$ $,k_{1}$ $,k_{2}$ $,k_{3}$ $,p_{1}p_{2}$		
	$a_{u,v}$$,b_{u,v}$, $\alpha_{\min},\alpha_{\max}$, $ratio\_u_{u,v},ratio\_v_{u,v}$	$\alpha_{u,v,0}$$,\delta_{u,v}$$,d_{i,j,k}$, $\alpha_{\min},\alpha_{\max}$, $ratio\_u_{u,v},ratio\_v_{u,v}$	$l_{1,n}$ $,l_{2,n}$ $,l_{3,n}$ $,l_{4,n}$
Memory Footprint	0.86 GB	3.42 GB	0.82 GB

**Table 2:** Computation time of the three methods (unit: ms).

Method	Platform	Computation Time	Data Transmission Time	Total Time
Low-Memory	CPU	43.15	-	43.15
	GPU	4.26	10.32	14.58
High-Memory	CPU	54.84	-	54.84
	GPU	4.15	6.48	10.63
Light-Plane	CPU	763.24	-	763.24
	GPU	64.72	10.27	74.99

**Table 3:** Planar RMS errors of the three methods (unit: mm).

Method	Low-Memory	High-Memory	Light-Plane
Planar RMS error	1.14	0.27	0.19

**Table 4:** Standard sphere accuracy of the three methods (unit: mm).

	Sphere 1		Sphere 2			
	Diameter	Error	Diameter	Error	Distance	Error
True Value	50.8019	-	50.8017	-	299.8513	-
Low-Memory	50.9349	0.1330	50.7446	0.0571	299.9740	0.1227
High-Memory	50.7091	**0.0928**	50.8398	**0.0381**	299.7463	**0.1050**
Light-Plane	50.9466	0.1447	50.8933	0.0916	299.9755	0.1242

## Data Availability

The original contributions presented in this study are included in the article. Further inquiries can be directed to the corresponding author.
